# Echotexture of recurrent laryngeal nerves: the depiction of recurrent laryngeal nerves at high-frequency ultrasound during radical thyroidectomy

**DOI:** 10.3389/fendo.2024.1356935

**Published:** 2024-09-12

**Authors:** Ziyue Hu, Man Lu, Zirui Jiang, Xu Wang, Wei Yang, Yuting Fan, Tingting Li, Lu Wang, Ting Wei, Quan Dai

**Affiliations:** ^1^ Department of Ultrasound, Sichuan Clinical Research Center for Cancer, Sichuan Cancer Hospital & Institute, Sichuan Cancer Center, Affiliated Cancer Hospital of University of Electronic Science and Technology of China, Chengdu, China; ^2^ School of Health Science, Purdue University, West Lafayette, IN, United States; ^3^ Department of Head and neck surgery, Sichuan Clinical Research Center for Cancer, Sichuan Cancer Hospital & Institute, Sichuan Cancer Center, Affiliated Cancer Hospital of University of Electronic Science and Technology of China, Chengdu, China

**Keywords:** recurrent laryngeal nerves, high-frequency ultrasound, radical thyroidectomy, peripheral nerve, recurrent laryngeal nerves invasion

## Abstract

**Introduction:**

To investigate the ultrasound characteristics of recurrent laryngeal nerves (RLNs) during radical surgery for thyroid cancer and to enhance the understanding of RLN ultrasound features.

**Methods:**

From October 2021 to December 2022, a prospective study was conducted involving 24 patients scheduled for bilateral thyroid surgery. Near the conclusion of the surgery, intraoperative ultrasonography of the RLN within the tracheoesophageal groove was performed using a 15-7 MHz transducer. The thickness and width of the RLN were measured during the procedure.

**Results:**

The internal architecture of the RLN was observed to consist of multiple hypoechoic, parallel, but discontinuous linear hyperechoic areas separated by bands. In the normal RLN group, the diameter of the RLN was relatively consistent, with thickness ranging from 2.20 to 2.71 mm (mean: 2.48 ± 0.14 mm) and width from 1.25 to 1.70 mm (mean: 1.45 ± 0.11 mm). Both weight and the body mass index (BMI) showed a statistically significant correlation with RLN thickness (Weight: r=0.544, P=0.001; BMI: r=0.605, P=0.001). The BMI also showed a statistically significant correlation with the RLN width (r=0.377, P=0.033). In the RLN invasion group, the width of invaded RLNs ranged from 1.9 to 2.3 mm (mean: 2.10 ± 0.11 mm), while the width of non-invaded RLNs ranged from 2.6 to 3.2 mm (mean: 2.93 ± 0.20 mm).

**Conclusions:**

Ultrasound effectively reveals the structural features of the RLN and enhances sonographers’ understanding of RLN characteristics.

## Introduction

**Figure 1:**
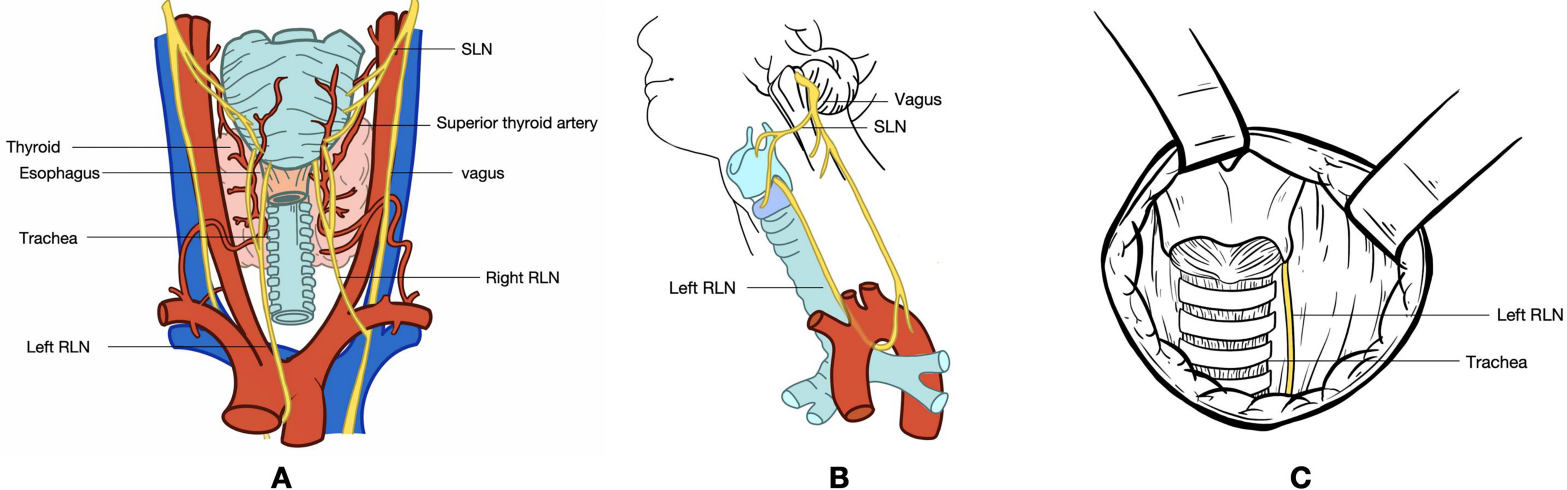
**(A)** Dorsal view of the thyroid gland. **(B)** Lateral view of the neck, illustrating the pathway of the vagus nerve. The vagus nerve originates from the medulla oblongata, exits the skull through the jugular foramen, and travels within the carotid sheath alongside the jugular vein. Before entering the carotid sheath or at the level of the greater horn of the hyoid bone, the vagus nerve gives rise to the superior laryngeal nerve. This nerve divides into medial and lateral branches, with the medial branch innervating the glottis and providing sensation in the throat, while the lateral branch innervates the cricothyroid muscle. The vagus nerve continues downward, giving off the left and right recurrent laryngeal nerves. The left recurrent laryngeal nerve loops around the aortic arch and ascends along the tracheoesophageal groove, taking a relatively long course. In contrast, the right recurrent laryngeal nerve encircles the subclavian artery, traverses the anterior part of the tracheoesophageal groove, and ascends obliquely upward and inward, following a shorter course. Both recurrent laryngeal nerves enter the larynx through the posterior aspect of the cricothyroid joint. **(C)** Intraoperative exposure of the RLN following thyroidectomy. The exposed segment of the RLN extends from the intersection of the RLN with the inferior thyroid artery branch to the lower edge of the cricoid cartilage, leading to the RLN’s entry into the larynx. RLN, recurrent laryngeal nerve; SLN, superior laryngeal nerve.

The recurrent laryngeal nerve (RLN), which is crucial for vocal fold mobility, is located in close proximity to the thyroid gland. The left vagus nerve descends along the carotid artery, enters the mediastinum, and passes anterior to the aortic arch. The left RLN (**Figure 1**), originating from the vagus nerve, loops medially under the aorta and ascends through the tracheoesophageal groove. Similarly, the right vagus nerve descends alongside the right common carotid artery, with the right RLN looping around the right subclavian artery and ascending along the tracheoesophageal groove (1, 2). In <1% of individuals, the right recurrent nerve originates directly from the vagus nerve at the level of the thyroid. Damage to the RLN can result in dysphagia and vocal cord hoarseness, while bilateral RLN injury can cause bilateral vocal cord paralysis, potentially leading to respiratory difficulties and posing a significant threat to patient life (3, 4). Studies have shown that thyroid surgery is the most common cause of RLN damage, with 5–10% of patients undergoing thyroid surgery experiencing RLN injury. This damage may result from thermal injury, traction, compression, cutting, or ligature during the surgical procedure (5).

High-frequency ultrasound is a highly effective diagnostic tool for examining superficial organs, as well as nerves, tendons, muscles, joints, and other soft tissues (6). It is widely used for the localization and visual assessment of peripheral nerves (7–9). Lu et al. demonstrated the practicality of ultrasonography in evaluating persistent peripheral nerve injuries 1 year after the Wenchuan earthquake, reinforcing its value for nerve evaluation (10). Additionally, studies have indicated that ultrasound-guided celiac plexus neurolysis is a safe and effective method for alleviating intractable upper abdominal pain in patients (11).

The structure of peripheral nerves typically appears as multiple parallel, yet disjointed, hypoechoic linear areas separated by hyperechoic bands in longitudinal views, and as multiple rounded hypoechoic areas within a homogeneous hyperechoic background in transverse views (12). However, research on the structural morphology of the RLN is relatively limited. The ultrasound images of the RLN presented in a few studies have shown inconsistent findings (12, 13). Some studies have described the RLN as a thin, hypoechoic structure, while others have depicted it as hyperechoic tissue (12, 13). This discrepancy creates confusion for sonographers, making it difficult to accurately identify the RLN. Moreover, there is a lack of studies providing a clear ultrasound visualization of the RLN in a living individual. In this study, we aimed to investigate the ultrasonographic structure of the RLN during radical thyroid cancer surgery, aiming to enhance the understanding of its ultrasonographic characteristics. Notably, this study provides the first clear observation of the RLN structure via ultrasound.

## Materials and methods

Between October and December 2022, we conducted a prospective study involving 24 patients diagnosed with papillary thyroid carcinoma (PTC) scheduled for bilateral thyroidectomy. The inclusion criteria were as follows: (i) patients diagnosed with thyroid cancer and undergoing radical surgery; (ii) patients aged ≥18 years; (iii) normal preoperative laryngeal findings; and (iv) normal preoperative voice. The exclusion criteria included: (i) pregnancy, significant coagulopathy, or severe systemic diseases; (ii) a history of thyroid, parathyroid, or neck dissection surgery, or microwave ablation (MWA) treatment; (iii) age <18 years; (iv) a history of neurological disorders or diseases, such as multiple sclerosis; (v) any preoperative benign vocal cord lesions or other voice disorders; and (vi) any preoperative pathological findings on videolaryngoscopy. All participating patients provided written informed consent. The study was approved by the Medical Ethics Committee of the Sichuan Cancer Hospital and Institute (SCCHEC-03-2017-008).

### Procedures

#### Thyroid surgery

Under general anesthesia, patients were positioned supine with their necks extended. Continuous nerve monitoring was employed throughout the surgery. A single arc-shaped incision, 2–4 cm in length, was made at the midline of the neck, just above the sternal notch, to perform the thyroidectomy. The skin, subcutaneous tissue, and platysma muscle were incised, and a skin flap was elevated, extending from the superior horn of the thyroid cartilage to the suprasternal fossae. Both ends of the flap were secured to the midline of the suprasternal fossae. The anterior neck muscles were retracted laterally, and the midline (linea alba cervicalis) was incised to expose the thyroid gland.

Neuromonitoring was performed using a commercial system (NIM-Response 2.0, Medtronic Inc., Minneapolis, MN), with surface electrode tubes used to record electromyography signals from the vocal cords (Medtronic Inc.). To minimize potential technical bias, endotracheal tube placement was typically handled by an experienced anesthesiologist. Intraoperative neuromonitoring (IONM) was conducted in accordance with the guidelines of the International Neural Monitoring Study Group (5).

#### US feature

As the operation neared completion, the RLN located in the tracheoesophageal groove became visible after the complete removal of the thyroid lobe containing the lesions. At this stage, the surgical area was rinsed with warm saline, and intraoperative ultrasonography was performed using a 15-7 MHz transducer of a Philips IU 22 ultrasound system (Philips, Amsterdam, The Netherlands). The probe, enclosed in a sterile sleeve and coated with acoustic gel, was placed over the RLN. During the ultrasound examination, the thickness and width of the RLN were measured. Each RLN was measured three times, and the average value was used for further analysis.

### Statistical analysis

Statistical analyses were conducted using SPSS 20.0 software (IBM, Armonk, NY, USA). Data with a normal distribution are presented as means ± standard deviations. Pearson’s correlation was used to assess the relationship between RLN thickness and width with patient weight, height, and BMI. Categorical data were analyzed using chi-square tests and are presented as percentages. Statistical significance was set at P<0.05.

## Results

### Clinical, surgical, and pathologic findings

All patients were pathologically confirmed to have PTC, with eight out of the 24 patients exhibiting surgical or pathological evidence of tumor invasion into the RLN. During surgery, RLN invasion was identified, and the nerve was separated from the tumor in these eight patients. The patients were categorized into two groups: those with normal RLN and those with RLN invasion. None of the patients presented with non-RLNs or symptoms indicative of RLN injury, such as vocal cord paralysis or hoarseness. Additionally, no incidents of signal loss were observed during IONM in any of the patients. Detailed patient information is provided in **Table 1**.

**Table 1:** Characteristics of the patients at baseline.

Patients	N=24
Age(years)	29.79 ± 6.00
Sex	
male	9
female	15
Weight (Kg)	58.50 ± 9.85
Height (cm)	164.63 ± 6.93
BMI (Kg/m^2^)	21.55 ± 3.17
Recurrent Laryngeal Nerves (RLN normal group)	
Thickness (mm)	2.48 ± 0.14
Width (mm)	1.45 ± 0.11
Left and Right RLN Thickness (RLN normal group)	
Left (mm)	2.48 ± 0.17
Right (mm)	2.47 ± 0.11
Left and Right RLN Width (RLN normal group)	
Left (mm)	1.45 ± 0.12
Right (mm)	1.46 ± 0.11

### Average parameter values of RLNs

**Figure 2:**
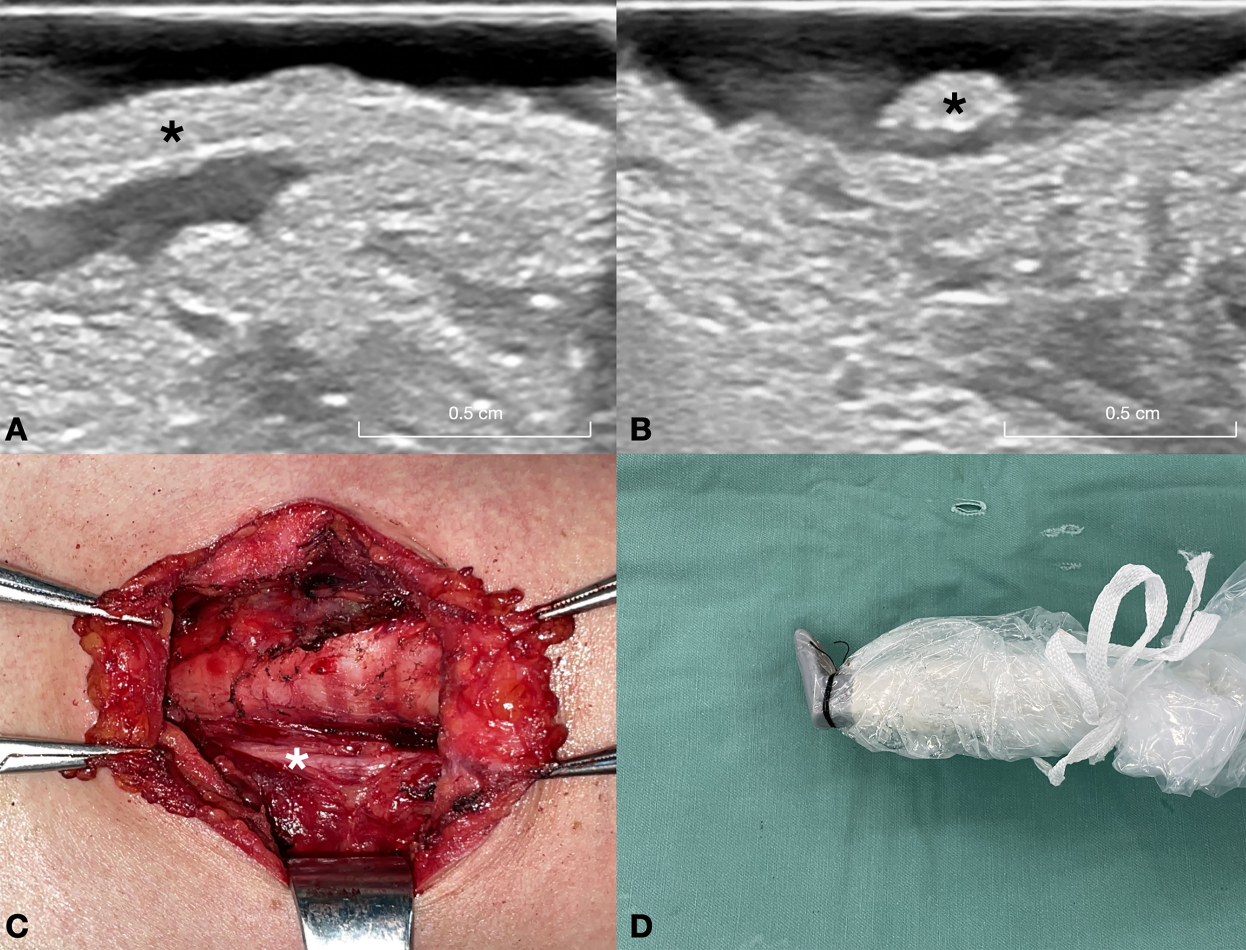
This figure depicts a 32-year-old female patient with a nodular goiter in the right lobe of the thyroid gland. **(A)** Long-axis ultrasound section of the normal RLN (asterisk). **(B)** Short-axis ultrasound section of the normal RLN (asterisk). **(C)** Intraoperative exposure of the normal RLN (asterisk) following thyroid gland excision. **(D)** Intraoperative ultrasound probe image of the normal RLN. A warm solution of physiological saline is administered into the surgical field, and the ultrasound probe is positioned above the RLN for imaging. RLN, recurrent laryngeal nerve.

A total of 48 RLNs were identified. In the normal RLN group, the RLN diameter was relatively consistent (**Figure 2**). The thickness of the RLNs ranged from 2.20 to 2.71 mm, with an average of 2.48 ± 0.14 mm, and the width ranged from 1.25 to 1.70 mm, with an average of 1.45 ± 0.11 mm. No significant differences were observed in the width and thickness of the RLN between the left and right sides (P>0.05). **Table 2** presents the correlation between patient characteristics and RLN thickness and width in the normal RLN group. Both weight and BMI showed a statistically significant correlation with RLN thickness (correlation coefficients: weight, r=0.544, P=0.001; BMI, r=0.605, P=0.001). A significant correlation was also found between the BMI and the RLN width (correlation coefficient: BMI, r=0.377, P=0.033).

**Table 2:** Association between characteristics of the patients and the thickness and width of RLN in RLN normal group.

Characteristics	Pearson Correlation	P- value
Thickness		
Age	0.169	0.354
Weight	0.544	0.001*
Height	0.016	0.93
BMI	0.605	0.001*
Width		
Age	-0.271	0.134
Weight	0.280	0.121
Height	-0.131	0.474
BMI	0.377	0.033*

The symbol "*" means that p is less than 0.05, and the difference is statistically significant.

**Figure 3:**
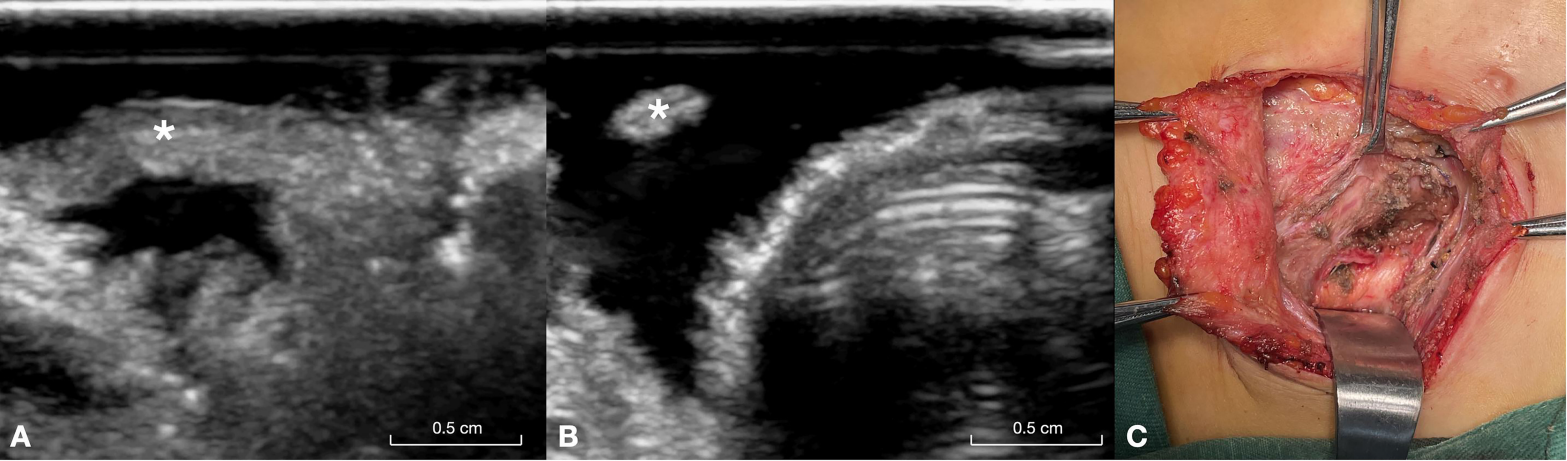
This figure illustrates a 26-year-old male patient with papillary carcinoma of the right lobe of the thyroid gland and invasion of the RLN. **(A)** Long-axis ultrasound section of the invaded RLN (asterisk), showing the distal end of the invasion site. **(B)** Short-axis ultrasound section of the invaded RLN (asterisk), highlighting the proximal segment of the invasion site, where the separated RLN appears very fine. **(C)** Intraoperative exposure of the invaded RLN (asterisk). RLN, recurrent laryngeal nerve.

In the RLN invasion group, the RLN diameter at the site of invasion was relatively smaller, whereas other parts of the RLN were thicker (**Figure 3**). The width of the invaded RLNs ranged from 1.9 to 2.3 mm, with an average of 2.10 ± 0.11 mm. In contrast, the width of other parts of the RLNs ranged from 2.6 to 3.2 mm, with an average of 2.93 ± 0.20 mm.

### The echotexture of recurrent laryngeal nerves

In the *in vivo* assessment of isolated RLNs using a 15-MHz probe, the internal structure of the nerves was characterized by multiple parallel, yet discontinuous, hypoechoic linear areas separated by hyperechoic bands in both the normal and invasion groups. The hypoechoic areas appeared sequentially, with an elongated and well-defined morphology. On transverse sections, multiple rounded hypoechoic areas were observed against a uniformly hyperechoic background.

## Discussion

Neuronal fascicles, which are the fundamental components of nerve structure, appear as hypoechoic on ultrasound. These fascicles consist of axon bundles interconnected by fine collagen strands, forming the endoneurium (14). Each fascicle is individually encased within a thin concentric layer of dense connective tissue known as the perineurium and is further bound to adjacent fascicles by the epineurium, a matrix of loose connective tissue (14). Sonographically, the homogeneous neuronal matrix within the fascicles may present as hypoechoic, while the surrounding connective stroma, which contains highly reflective adipose tissue, tends to appear hyperechoic. The number of fascicles is reportedly correlated with nerve size, with smaller nerves, such as those found in the extremities, often containing a single fascicle (15). Ultrasound has been extensively utilized in nerve studies, including observations of the sciatic and carpal nerves. In our study, the RLN was clearly visualized using warm physiological saline as an acoustic window. The RLN appeared as an oval, hypoechoic structure encased in hyperechoic tissue. This represents the first *in vivo* observation of the distinct ultrasonic structural characteristics of the RLN.

In the normal RLN group, no significant differences were observed in the width and thickness between the left and right RLNs. Our findings demonstrated a significant correlation between RLN thickness and factors, such as body weight and BMI. Additionally, RLN width was closely associated with BMI. Similarly, Wu et al. reported that RLN diameter was positively correlated with body size, as determined by weight, height, and BMI (16). In the RLN invasion group, the RLN diameter was reduced at the site of compression, while the distal end of the nerve became thicker, likely due to edema. Wu et al., using a Castroviejo caliper to measure the maximum RLN diameter, documented an average RLN diameter of 1.71 mm (16).

In our study, the thickness of the RLNs ranged from 2.20 to 2.71 mm, with an average of 2.48 ± 0.14 mm, and the width ranged from 1.25 to 1.70 mm, with a mean of 1.45 ± 0.11 mm. It is important to acknowledge potential discrepancies in measurement methods, as our study utilized ultrasonic measurements, which may introduce certain data variances when compared to the study by Wu et al. We observed that the RLN diameter at the site of invasion was relatively smaller, while other parts of the RLN were thicker in the RLN invasion group. This observation is consistent with the findings of Serpell et al., who reported an approximate 1.5-fold increase in RLN diameter during lobectomy procedures (17). This phenomenon can be attributed to intraoperative nerve swelling.

In conclusion, this study represents the first application of intraoperative ultrasound to investigate the RLN. Ultrasound provides a distinct visualization of the structural characteristics of the RLN, enhances the understanding of this nerve among ultrasound practitioners, and facilitates the preoperative ultrasound assessment of the RLN.

## Data Availability

The raw data supporting the conclusions of this article will be made available by the authors, without undue reservation.

## References

[1] Ardito G Revelli L D'Alatri L Lerro V Guidi ML Ardito F. Revisited anatomy of the recurrent laryngeal nerves. Am J Surg. (2004) 187:249–53. doi: 10.1016/j.amjsurg.2003.11.001 14769313

[2] Lee MS Lee UY Lee JH Han SH. Relative direction and position of recurrent laryngeal nerve for anatomical configuration. Surg Radiol Anat. (2009) 31:649–55. doi: 10.1007/s00276-009-0494-y 19326038

[3] Chiang FY Lu IC Kuo WR Lee KW Chang NC Wu CW. The mechanism of recurrent laryngeal nerve injury during thyroid surgery–the application of intraoperative neuromonitoring. Surgery. (2008) 143:743–9. doi: 10.1016/j.surg.2008.02.006 18549890

[4] Snyder SK Lairmore TC Hendricks JC Roberts JW. Elucidating mechanisms of recurrent laryngeal nerve injury during thyroidectomy and parathyroidectomy. J Am Coll Surg. (2008) 206:123–30. doi: 10.1016/j.jamcollsurg.2007.07.017 18155577

[5] Randolph GW Dralle H International Intraoperative Monitoring Study Group Abdullah H Barczynski M Bellantone R. Electrophysiologic recurrent laryngeal nervemonitoring during thyroid and parathyroid surgery: international standards guideline statement. Laryngoscope. (2011) 121 (Suppl 1):S1–16. doi: 10.1002/lary.21119 21181860

[6] Kaplan PA Matamoros A Jr Anderson JC. Sonography of the musculoskeletal system. AJR Am J Roentgenol. (1990) 155:237–45. doi: 10.2214/ajr.155.2.2115246 2115246

[7] Abraham A Izenberg A Dodig D Bril V Breiner A. Peripheral nerve ultrasound imaging shows enlargement of peripheral nerves outside the brachial plexus in neuralgic amyotrophy. J Clin Neurophysiol. (2016) 33:e31–3. doi: 10.1097/WNP.0000000000000304 27749462

[8] Ng ES Vijayan J Therimadasamy A Tan TC Chan YC Lim A. The added value of preoperative ultrasonography of the ulnar nerve: an observational study. Muscle Nerve. (2010) 42:613–4. doi: 10.1002/mus.21800 20878743

[9] Samarawickrama D Therimadasamy AK Chan YC Vijayan J Wilder-Smith EP. Nerve ultrasound in electrophysiologically verified tarsal tunnel syndrome. Muscle Nerve. (2016) 53:906–12. doi: 10.1002/mus.24963 26562220

[10] Lu M Wang Y Yue L Chiu J He F Wu X. Follow-up evaluation with ultrasonography of peripheral nerve injuries after an earthquake. Neural Regener Res. (2014) 9:582–8. doi: 10.4103/1673-5374.130095 PMC4146238 25206859

[11] Wang L Lu M Wu X Cheng X Li T Jiang Z. Contrast-enhanced ultrasound-guided celiac plexus neurolysis in patients with upper abdominal cancer pain: initial experience. Eur Radiol. (2020) 30:4514–23. doi: 10.1007/s00330-020-06705-z 32211966

[12] Silvestri E Martinoli C Derchi LE Bertolotto M Chiaramondia M Rosenberg I. Echotexture of peripheral nerves: correlation between US and histologic findings and criteria to differentiate tendons. Radiology. (1995) 197:291–6. doi: 10.1148/radiology.197.1.7568840 7568840

[13] He Y Li Z Yang Y Lei J Peng Y. Preoperative visualized ultrasound assessment of the recurrent laryngeal nerve in thyroid cancer surgery: reliability and risk features by imaging. Cancer Manag Res. (2021) 13:7057–66. doi: 10.2147/CMAR.S330114 PMC8439442 34531684

[14] Bloom W Fawcett DW. A textbook of histology. 11th ed. Philadelphia, pa: saunders (1986) p. 335–7.

[15] Ham AW Cormack DH. Histology. 8th ed. Philadelphia, pa: lippincott (1979) p. 527–9.

[16] Wu KT Chan YC Chou FF Wu YJ Chi SY. Association between recurrent laryngeal nerve calibre and body figure: A preoperative tool to assess thin-diameter nerves in thyroidectomy. World J Surg. (2020) 44:3036–42. doi: 10.1007/s00268-020-05549-4 32385681

[17] Serpell JW Lee JC Yeung MJ Grodski S Johnson W Bailey M. Differential recurrent laryngeal nerve palsy rates after thyroidectomy. Surgery. (2014) 156:1157–66. doi: 10.1016/j.surg.2014.07.018 25444315

